# Fluid Dynamics of a Spouted Bed with a Fountain Deflector

**DOI:** 10.1021/acsomega.5c09057

**Published:** 2025-12-15

**Authors:** Lucas Caiafa Cardoso Reis, Ana Carolina Tavares Silva, Iara Hernandez Rodriguez, Isabele Cristina Bicalho

**Affiliations:** Chemical Engineering and Materials Department, 67739 Federal University of Lavras, Lavras, Minas Gerais 37200-000,Brazil

## Abstract

Spouted beds can be applied in various operations; even so, they have limited industrial use. Therefore, internal accessories and variations in their configuration have been studied. In this study, an uncommon device known as a fountain deflector was inserted into a conventional conical spouted bed to evaluate its influence on particle elutriation and on the hydrodynamics of the bed operating with dried papaya seeds. The physical characterization of the particles was carried out considering both their individual properties and those of the porous medium formed by them. The main characteristic of the seeds is their low apparent density (610 kg/m^3^) and the formation of a bed with a high porosity (0.73). The fluid dynamic curves were obtained for the equipment without a deflector and with the deflector positioned at 15 cm in relation to the conical base of the bed. It was noticed that the presence of the deflector reduced particle elutriation, without significantly affecting the values of the fluid dynamic parameters. The CFD simulation results showed the particle retention effect of the deflector, where it limits the maximum spoutable bed height and reduces the drag. A reduction in particle size distribution was observed when operating with the deflector at high air velocities. Therefore, this study contributed to the evaluation of a rarely used device that demonstrates potential for use in industrial applications. The findings suggest that the deflector can act by maintaining a stable spouting and reducing particle elutriation, also showing potential to promote particle breakage.

## Graphical abstract

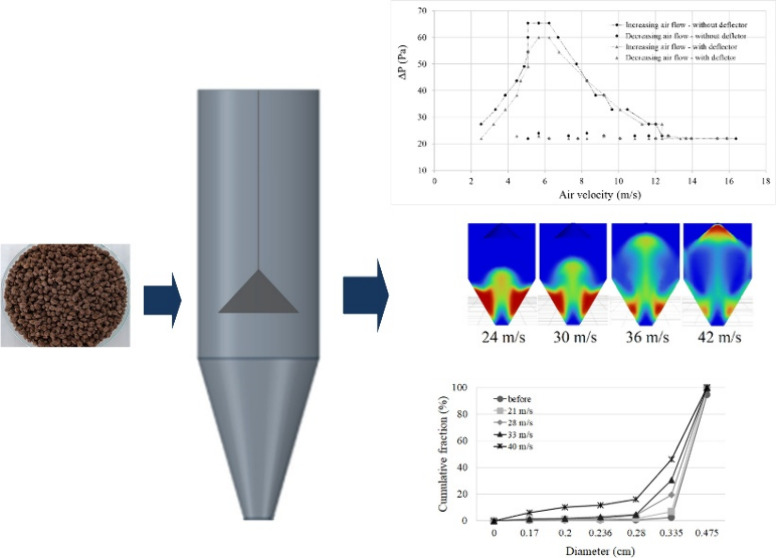

## Introduction

The main characteristic of the spouted bed is the cyclical movement of particles, which promotes a high contact between the solid and fluid phases, i.e., a high degree of mixture and heat and mass transfer rates.^1^ The equipment is simple and of easy maintenance, requires low initial and operational investments, and therefore stands out as an alternative for application in various processes, such as seed and paste drying, particle coating, inoculation, granulation, pyrolysis, among others.^2–5^

The original model developed by Mathur and Gishler^6^ consisted of a cylindrical body with a small conical base, without the presence of any internal accessory. This original configuration led to some restrictions in applications, for example, when operating with irregular, fine, sticky particles, or particles with a wide size distribution, where it conducted to unstable flows, especially on a large scale.^7^

Alternatives to reduce instabilities in the spouted bed and expand its application include geometric modifications and the insertion of internal devices such as mechanical agitators and draft tubes.^8–11^

Draft tubes and plates are devices inserted to the bed in order to guide the particle distribution. The insertion of these devices positively affects the hydrodynamics stability, solid circulation, spouting velocity and pressure drop. It is possible to achieve better control over the circulation of solids through the spout–annulus region and to constrain spout deflection, which assists in obtaining a stable spouting regime and enables the operation of the bed with a wider range of particle sizes and shapes.

However, it is also highlighted in the literature that the use of these accessories can cause a decrease in the degree of mixing and gas-particle contact, which would lead to lower mass and heat transfer coefficients.^10,12–18^

A device called fountain confiner has been studied in recent years to stabilize the flow of fine and ultrafine particles with a wide size distribution in a spouted bed.^18–20^ The accessory developed by Altzibar et al.^16^ with the aim of preventing the dragging of fine particles, is formed by a body in the shape of a cylindrical tube, and a closed upper cover in a conical shape. The accessory is positioned at the top central part of the bed. When the spouting process is initiated, the spouting is confined in the body of the confiner, and upon reaching the top, the air flow from the fountain is directed downward, passing between the bottom of the confiner and the surface of the bed. As a result, particles cannot be dragged out of the bed and fall back onto its surface.

The use of fountain confiners has shown great advantages for the operation of spouted beds with fine particles, such as reducing the operating pressure drop, preventing the elutriation of solids and controlling the maximum height of the spout in addition to uniformizing the circulation of particles, maximizing the gas–solid contact.^18,20–22^

Sukunza et al.^22^ reported good stability and high efficiency in the drying process of fine and ultrafine particles in a conical spouted bed using a fountain confiner combined with a draft tube and a particle feeder. According to the authors, as the confiner forces the gas to circulate inside the device, the residence time of the gas in the drying compartment increases, favoring gas–solid contact, which leads to large improvements in drying efficiency.

Another device that can assist in spouted bed operation and is rarely mentioned in the literature is the fountain deflector. According to Mathur and Epstein,^1^ the device is efficient in controlling the height of the spout and helps in its centralization by increasing the symmetry in the distribution of particles as they return to the bed. The accessory called a fountain deflector is similar to a fountain confiner; however, this device does not have a cylindrical body part, being formed only by a conical or hat-shaped body. When bed operation starts, the jet is not confined within the body of the device as is the case with the fountain confiner. It acts as a physical barrier that redirects particles back to the bottom of the bed, mainly helping to control the fountain height.

It is important to emphasize that, to date, no studies have reported the use of this accessory, known as a fountain deflector, in spouted beds. Based on the studies cited, most modifications of spouted beds are aimed at improving spout regime stability and reducing spout deflection.^12,23^ There are studies reporting the performance of internal accessories in reducing elutriation during the processing of fine particles, that experience severe elutriation.^20,24,25^ The fountain deflector is a simple device that shows potential to improve spouted bed operation, particularly in processes involving smaller and/or lighter particles at high gas velocities, where a high fountain is formed and some particle elutriation from the bed may occur. It could be particularly useful, for example, in seed drying processes, especially for low-density seeds, which tend to become even lighter as their moisture content decreases during drying,, thus favoring their elutriation from the bed.

Also, according to Mathur and Epstein,^1^ the fountain deflector can enhance particles spread in the annular region, minimize spout oscillations, and if the process is carried out at high air velocities, the device could also act as a particle breaker. Depending on the material and process, high gas velocities could lead to high particle attrition caused by the contact between solids and the interior of the device. This limitation could be particularly relevant in processes involving fragile or valuable solids, such as pharmaceutical granulation, catalytic processes, drying of granulated materials or very fragile agregates.^2,26,27^

As observed, several modifications of spouted beds have expanded its application possibilities with a greater range of particle sizes and shapes, however none of the adaptations have yet been able to completely resolve its limitations. Therefore, the search for solutions that guarantee the maintenance of a stable fluid dynamic regime in large-scale spouted beds is fundamental.

In view of the above, the objective of this work was to investigate experimentally and through computational simulations the performance of the fountain deflector in a conventional conical spouted bed operating with light particles of size around 5 mm and its applicability to reduce particle elutriation.

## 1 Materials and Methods

### 1.1 Raw Material and Characterization

In this study, dried papaya seeds without exotesta, a protective film of the pores, were used as particulate material (Figure 1). Previous studies by the same research group^5^ showed that such seeds have properties favorable to elutriation in the spouted bed, therefore, they would allow a better evaluation of the contribution of the deflector in the stabilization of the flow.

**Figure 1:**
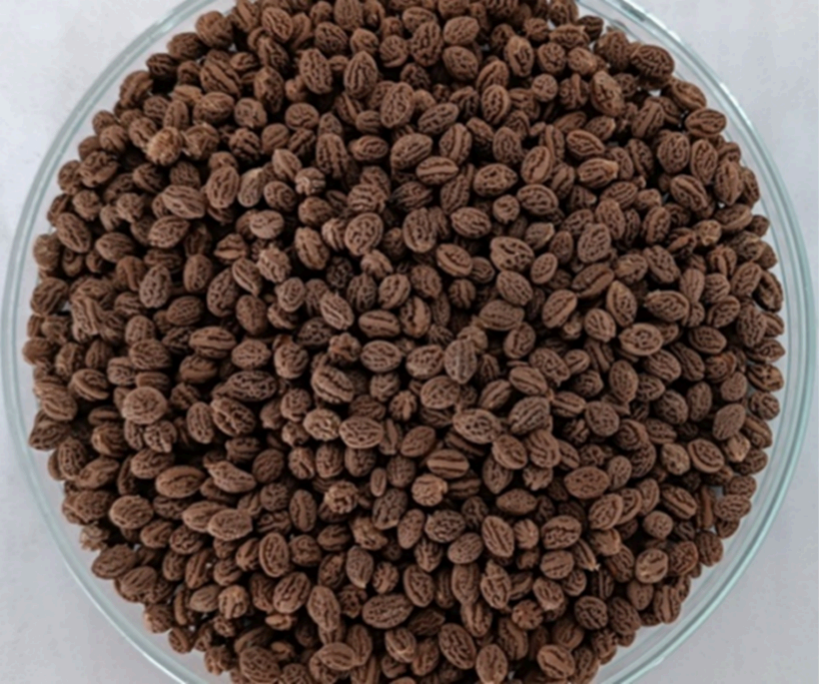
Dried papaya seeds.

The seeds were characterized according to their physical properties and the porous medium formed by them, and the following properties were determined: mean diameter, sphericity, apparent density, bulk density, bulk porosity, static and dynamic angle of repose. All analyses were performed in triplicate, and the average results and standard deviation were obtained. The seeds were also classified according to the categories defined by Geldart.^28^

The mean diameter and sphericity of the seeds were determined by image analysis using FIJI software version 2.15. For this purpose, samples of 200 seeds were collected and arranged in a white sheet. After this, the seeds were photographed and their images were processed and analyzed using image analysis software.

The apparent density of the seeds was determined by liquid pycnometry. The bulk density and porosity were determined from the beaker deposition technique, as procedure detailed by de Lima Santos et al.^5^

To determine the static angle of repose, a funnel with a smaller base diameter of 0.03 m and a larger base with a diameter of 0.15 m was used, positioned at a height of 0.19 m from a flat bench. The seeds were poured and accumulated forming an angle in relation to the horizontal base. The front view of the pile of particles was photographed and the angle formed was calculated using the FIJI software.

To determine the dynamic angle of repose, the rotating drum method was used. A drum with a transparent side and a marking in the center was filled to half its volume with seeds and placed on 2 moving rollers. With the drum rotating, video images were captured for 5 min, and after the end, 5 frames of the recording were randomly selected and analyzed to measure the angle formed by the seeds in relation to the central reference line.

### 1.2 Experimental Unit

The experimental unit (Figure 2) is constructed of stainless steel, equipped with a 7.5 HP blower (1), a gate valve to control the air flow, and an orifice plate (2) equipped with pressure sensors to measure the air velocity. The bed (3) has a cylindrical column, open at the top, with a diameter of 0.256 m and a height of 0.508 m, equipped with a transparent acrylic display which allows visual observation of the bed. The base of the bed is formed by a cone trunk with a larger diameter equal to that of the cylinder, a smaller diameter of 0.05 m, a height of 0.176 m and an opening angle of 60°. The diameter of the air inlet orifice is the same diameter of the cone base. The pressure was measured at the air inlet below the bed, which was open to the atmosphere at the top. Bed and orifice pressure drop were measured using differential pressure transducers. The transducers send the 4–20 mA signal to a data logger which is connected to a computer where the data are registered. Prior to the experiments, the sensors were calibrated against a U-tube manometer used as a reference standard. For the operation of the system, a frequency inverter (4) is controlled by a computer using a software with an interface (5), which also allows data acquisition.

**Figure 2:**
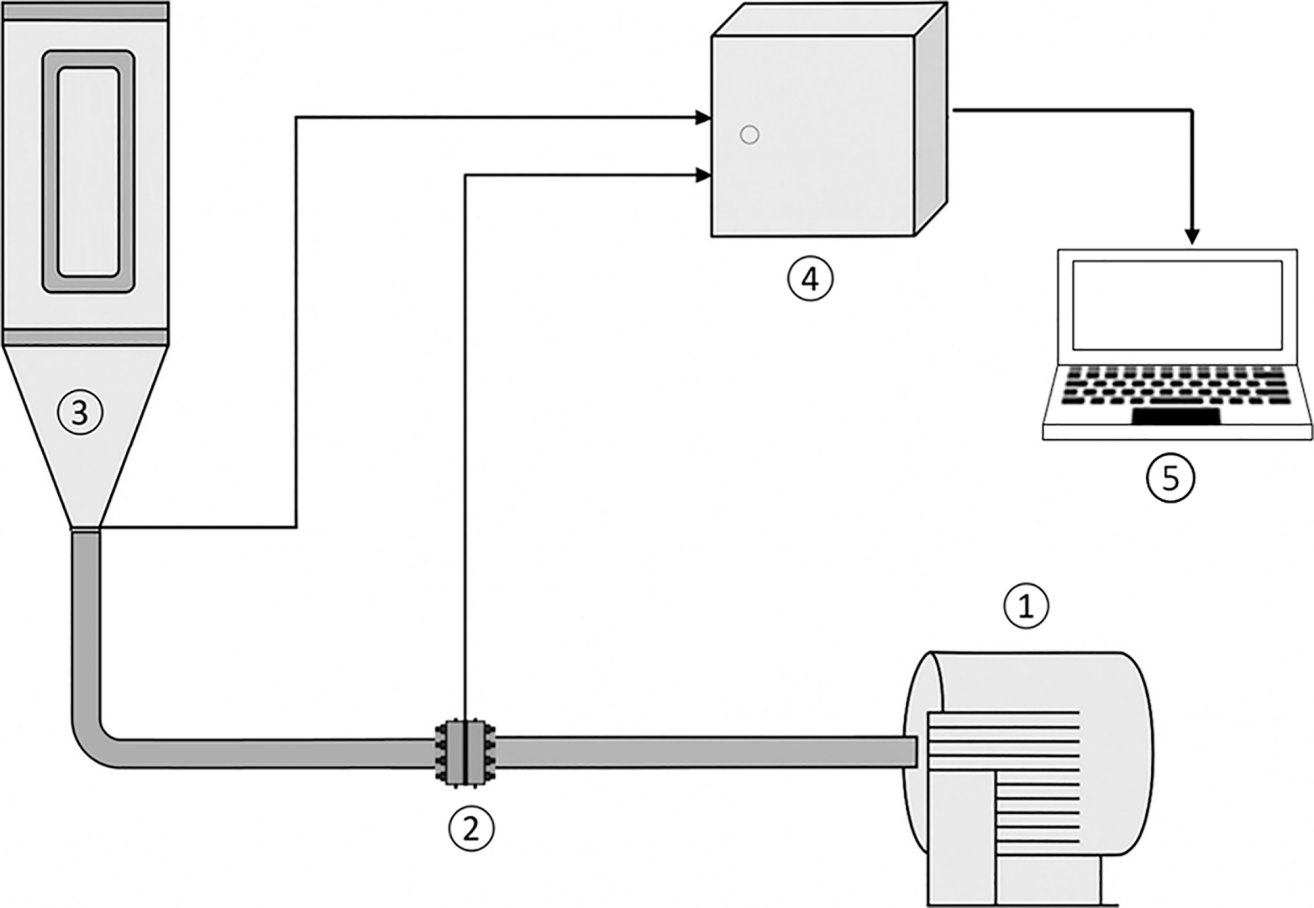
Experimental unit.

### 1.3 Fountain Deflector

The fountain deflector (Figure 3) was designed based on the geometry of the fountain confiner used by Altzibar et al.^16^ and the deflectors presented by Mathur and Epstein.^1^ The accessory resembles the fountain confiner; however, it is formed only by a conical body with a diameter of 0.18 m and a height of 0.07 m.

**Figure 3:**
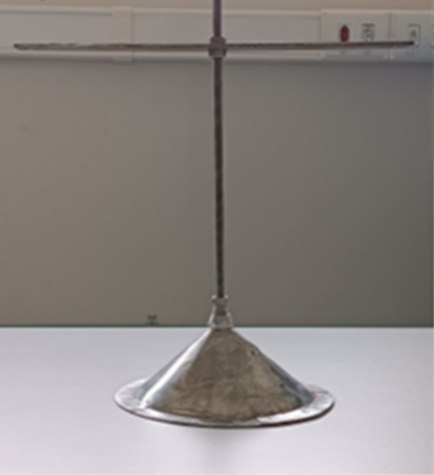
Fountain deflector.

The piece was designed with a conical shape to facilitate particle movement and redirection toward the top of the annular region, while preventing solid deposition by allowing particles to slide back into the bed. The device is constructed of stainless steel and is attached to a fixing rod, which allows adjustment of its height/position inside the bed.

The purpose of using such internal accessory is to guarantee the stabilization of the flow of relatively large and light particles in a spouted bed, which would normally lead to unstable flows or be elutriated.

### 1.4 Fluid Dynamics Characterization of the Spouted Bed

The fluid dynamic characterization of the seeds in the spouted bed was performed by surveying the characteristic curve of the equipment with the fountain deflector positioned at a height of 0.15 m in relation to the conical base of the bed and the experiment was repeated without the deflector (Figure 4).

**Figure 4:**
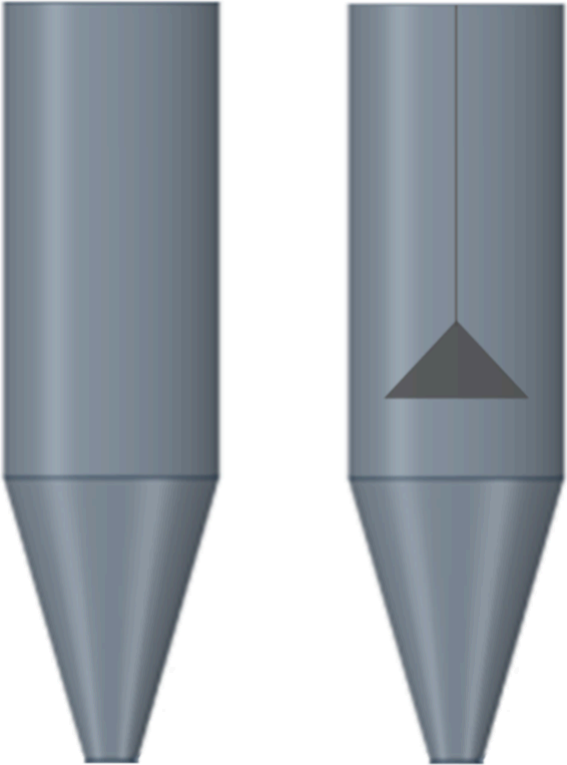
Schemes of spouted bed without and with fountain deflector.

To obtain the curve, 400 g of dried papaya seeds was added to the bed, which corresponds to a bed height of 0.141 m, and then air was injected through the inlet orifice. The air flow rate was gradually increased to the maximum capacity of the blower, and the corresponding values of pressure drop was recorded using data acquisition software. When the maximum capacity of the blower was reached, the inverse procedure was initiated, that is, the air velocity was gradually reduced and the pressure drop values were measured. As the test was carried out, the evolution of the bed was visually observed and any instability or elutriation was registered.

### 1.5 Comminution of Particles in the Bed with the Deflector

For the experimental particle fragmentation tests, the deflector was positioned at a height of 0.06 m in relation to the conical base of the bed. The bed was operated with a seed load of 0.075 kg for 30 min at air velocities of 21, 28, 33, and 40 m s^–1^. The initial particle size distribution of the seeds was obtained by sieving, as well as their distribution at the end of the operating time for each of the conditions tested.

### 1.6 Numerical Procedure

Numerical simulations were performed allowing the visualization of the fluid dynamic behavior of the spouted bed with the deflector. For this purpose, Ansys R21.0 *Academic* package was used and the procedure adopted is described below.

#### 1.6.1 Geometry

The three-dimensional geometry of the spouted bed with the fountain deflector positioned at a height of 0.15 m in relation to the conical base of the bed was constructed, Figure 5. The cylindrical part of the bed was constructed with a height of 0.21 m, coinciding with the top of the deflector, in order to reduce the time and computational costs of the simulations. The other measurements were faithful to the original measurements of the equipment.

**Figure 5:**
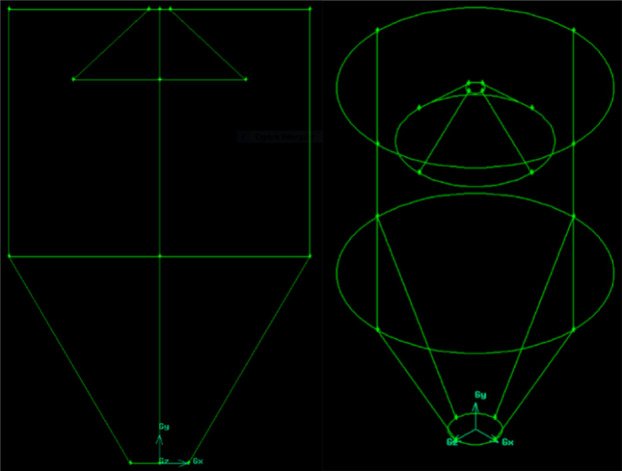
3D geometry of the spouted bed with a fountain deflector.

#### 1.6.2 Meshes and Independence Test

It is known that the flow inside the spouted bed is quite complex and demands a long simulation time, therefore, a mesh independence test was carried out with the objective of finding a mesh with a reduced number of cells, but which did not compromise the quality of the results obtained. Three meshes with 217k, 334k and 408k tetrahedral cells were created. In the independence test, meshes were evaluated for quality parameters such as aspect ratio, skewness and orthogonal quality and presented good results. The effect of mesh size on the simulation results was observed through the analysis of the fluid dynamics curves simulated, as done by de Lima Santos et al.^5^ Thus, the results obtained were compared to choose the best mesh, the one which leads to the least computational effort.

#### 1.6.3 Physical Parameters and Computations

All the physical parameters of the problem, such as wall, interior, inlet and outlet, and internal walls such as the deflector, were previously defined. In the simulations of the bed without the presence of the deflector, the deflector walls were defined as interior. The experimental values obtained for the mean diameter, apparent density and porosity of the papaya seeds were used to define the properties of the solid phase. In addition to the physical definitions, other parameters selected to perform the calculations are shown in Table[Table tbl1]. The models and parameters used in the simulations were based on previous studies from the literature and on earlier simulations of the same system.^5,29,30^

**Table 1:** Boundary Conditions and Parameters Used in Simulations

model	Eulerian multiphase: implicit
interaction fluid–particle	Gidaspow
granular particle	granular viscosity: Syamlal-Obrien
	granular bulk viscosity: Lun et al. 1984 ^31^
	frictional viscosity: Not enabled
	granular temperature: algebraic
	solids pressure: Lum et al. 1984 ^31^
	radial distribution: Lum et al. 1984 ^31^
	modulus of elasticity: derivative
turbulence model	K-epson pattern
coupling pressure–velocity	SIMPLE
spatial discretization	gradient: least squares cell based
	pressure: PRESTO
	momentum: first order upwind
	volume fraction: first order upwind
relaxation parameters	pressure: 0.3
	density: 1
	body forces: 1
	momentum: 0.1
	volume fraction: 0.5
	granular temperature: 0.2
	turbulent kinetic energy: 0.8
	turbulent dissipation rate: 0.8
	turbulent viscosity: 1
residual convergence criteria	10^–3^
time step	10^–4^
simulation time	until 4 s

The simulations were performed on a notebook with a quadcore Intel Core i7–7500U 2.70 GHz processor with 8 GB of RAM and 1 TB SSD, an INTEL Core i7–8700 3.2 GHz computer with 16GB of RAM and 240GB SSD PRO Certo PC, and 20 Dell workstation computers with 6 Intel Xeon w-1350 4.5 GHz processors with 32 GB of RAM and 512 GB SSD.

It is worth noting that the particle selected for the experimental and/or numerical studies presented uniform characteristics, which did not vary over time, and maintained its structure (did not undergo disintegration) in the operation for the analyzed flows. Furthermore, such particle is light and presents characteristics favorable to elutriation and obtaining unstable regime flows in a spouted bed, therefore, allowing a better evaluation of the contribution of the deflector in the stabilization of the flow.

## 2 Results and Discussion

### 2.1 Seeds and Particle Bed Characterization

The average values of the properties measured for the dried papaya seeds and for the porous bed formed by them are shown in Table 2.

**Table 2:** Characterization of Papaya Seeds and the Porous Bed

parameter	value
apparent density (kg m^–3^)	610 ± 30
mean diameter (m)	0.0045 ± 0.00003
sphericity (%)	84.50 ± 4.7
bulk density (kg m^–3^)	160 ± 10
porosity (%)	73 ± 0.01
static angle of repose (°)	34.42 ± 3.33
dynamic angle of repose (°)	38.53 ± 2.41

The properties obtained in this work, for dried papaya seeds, as apparent density, mean diameter and sphericity, are similar to the values measured by de Lima Santos et al.^5^ and Chielle et al.^32^ Dried papaya seeds were classified as type D particles in the Geldart diagram,^28^ large and spoutable particles, appropriate for application in the spouted beds.

The sphericity value measured was high, indicating the proximity of a sphere shape for the seed. The low value obtained for bulk density and high value for porosity indicate that the seeds present low compaction when arranged in a bed. The values of the static and dynamic angle of repose obtained, which were 34.42° and 38.53°, respectively, indicate that the dried papaya seeds present good flowability. These results are in agreement with those found in the literature.^5,32^

The parameters presented in this section are important for a better understanding of the flowability capacity of the particulate material studied and for predicting its behavior in the spouted bed.

### 2.2 Characteristic Curve

Figure 6 shows the fluid dynamic curves of the bed, which shows the relationship between the pressure drop (Δ*P*) in the bed and the air velocity, in round-trip conditions, with and without the presence of the fountain deflector.

**Figure 6:**
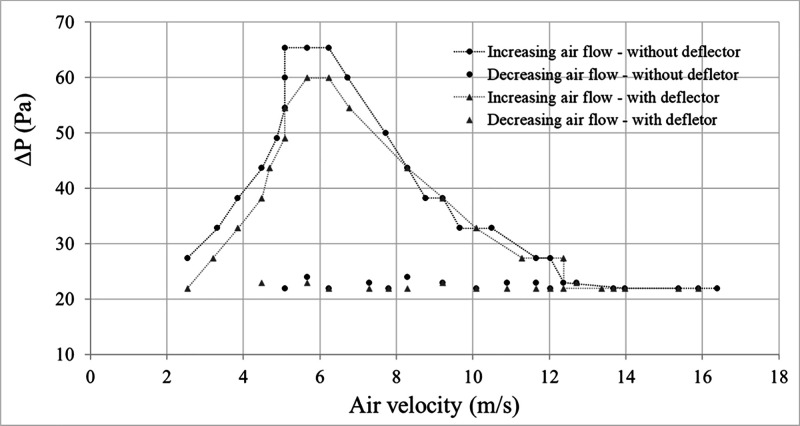
Experimental fluid dynamic curves.

Analysis of Figure 6 indicates that the fountain deflector did not affect the typical behavior expected for the variation of pressure drop with air velocity in spouted beds. At the initial section of the curve, an increase in pressure drop is observed corresponding to the rise in airflow velocity. In this region the bed remains fixed, with the air only percolating between the particles. As the air velocity increases, an internal cavity is developed which will push the packed bed above it, so the pressure drop will continue to rise until it reaches a maximum. As this internal cavity grows significantly in comparison to the packed bed above, the bed’s resistance to flow decreases and the pressure drop begins to fall. Subsequently, bed expansion occurs, leading to the opening of its upper surface. Such opening results in an abrupt pressure drop, which signals the formation of the spouting regime. Beyond this point, additional increases in air velocity result solely in an increase in spout height, with no further change in pressure drop.

The minimum spouting velocity (*V*_*mj*_) and the maximum pressure drop (Δ*P*_*max*_) were identified based on the fluid dynamic curves (Figure 6) and visual inspection of the seeds behavior inside the equipment during testing. Notably, the use of the deflector did not result in any considerable changes in the values of these fluid dynamic parameters or in the particle circulation pattern.

The fluid dynamic curve suggests a slight reduction in the maximum spouting pressure drop (Δ*P*_max_). with the insertion of the accessory into the system, which could be attributed to experimental uncertainty. The minimum spouting velocity (*V*_*mj*_) was very similar for the bed with and without the deflector, remaining around 13 m s^–1^. This result could suggest that stable bed operation might be attained at similar velocities, whether or not the deflector is used.

The fluid dynamic results obtained in this study for the bed with the fountain deflector are similar to those reported in other studies using a similar accessory, known as a fountain confiner.^7,19,33^ Altzibar et al.^16^ introduced the fountain confiner as a means to enhance the stability of spouted bed processes dealing with fine and ultrafine particles. The fountain confiner enables stable spouted flow of particles that would otherwise be elutriated in a conventional bed. They observed that the use of the fountain confiner combined with a draft tube reduced particle loss (or elutriation) by 60% to 70%, depending on the type of draft tube used, without affecting the minimum spouting velocity. In addition, the authors reported a reduction of up to 14% in the operating pressure when the confiner was used. For the authors, the reason for the decrease in pressure lies in the increase in bed stability with the confiner.

The contribution to the stability of the flow is certainly an aspect that stands out regarding the use of the fountain deflector. It was observed that the deflector essentially prevented particle elutriation. This is because the device acts as a physical barrier, by limiting the height of the spout, redirecting particles back to the top of the annular region and creating highly stable, well-defined flow patterns.

De Lima Santos et al.^5^ employing the same system used in this study, reported observing flow instabilities in dried papaya seeds. According to the authors, the formation of the spout was accompanied by the pneumatic transport of a significant portion of the material. In this study, no such behavior was observed with the use of the deflector. From visual observation it was possible to verify well-defined flow patterns generated in the region between the bed’s base and the fountain deflector. It is important to note that the fountain geometry was not altered; only the height of the spout was restricted by the deflector. Also, a central region on the bed with a clear definition of the spout was observed, resulting in a stable spouted flow, without significantly altering the expected fluid dynamics of the spout.

These results imply that the deflector allows better control of particle circulation and contributes to enhanced particle retention in the bed, making it advantageous for systems with high elutriation where minimizing particulate material loss is crucial. This feature makes the deflector particularly useful in applications where the bed operates with smaller and/or lighter particles, which would normally be elutriated, thereby allowing them to remain within the equipment.

### 2.3 Particle Fragmentation Analysis

The results of particle fragmentation in the operation with the fountain deflector are shown in Figure 7. This figure presents the size distribution of the seeds before they were added to the bed as well as the distributions after 30 min of operation in the bed with the deflector, at different air velocities (21, 28, 33, and 40 m/s).

**Figure 7:**
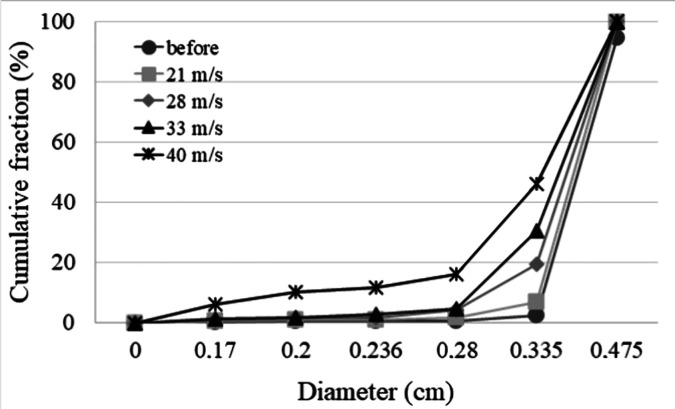
Cumulative particle size distribution curves for the seeds in the bed with the deflector.

Figure 7 shows that with increasing the air velocity a deviation of particle size distribution curves to the left side is observed, indicating an increasing number of smaller particles. This indicates the particle fragmentation observed during bed operation with the deflector.

For all air velocities analyzed, stable spouting was achieved, and collisions of particles with the inner surface of the deflector were observed. At air velocities above 28 m s^–1^, the effect becomes more pronounced, which can be attributed to greater particle fragmentation resulting from repeated impacts and intensified friction between the seeds and the inside of the deflector. At lower air velocities, although the seeds also reached the deflector, the lower velocity reduced the severity of impact and friction, resulting in practically no particle breakage.

Thus, the results indicate that the deflector could be regarded as a strategic accessory for improving spouted bed operation, ensuring greater bed stability when handling materials with challenging granulometric and density properties, without significantly altering the system’s fluid dynamics. In addition, the findings indicate the fountain deflector’s potential role in promoting particle fragmentation.

### 2.4 Numerical Results

#### 2.4.1 Mesh Independence Test

The mesh independence test was conducted by simulating the fluid dynamic curve of the spouted bed with the fountain deflector at different inlet air velocities (4, 8, 12, 24, and 30 m/s), as shown in Figure 8.

**Figure 8:**
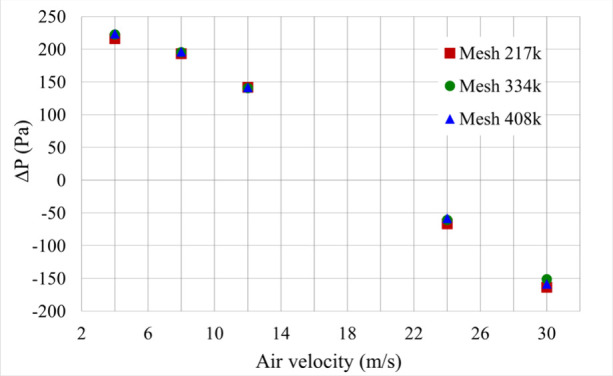
Results of the mesh independence test.

Figure 8 reveals that all three meshes exhibited similar behavior, with pressure drop values being equal or nearly identical within the same air velocity range. Therefore, the 334k mesh was selected for the subsequent simulations due to its intermediate number of cells and lower computational cost compared to the 408k mesh. The characteristic flow patterns of the spouted bed were observed through the analysis of solid volume fraction contours generated at each air velocity used in the simulation of the fluid dynamic curve in Figure 9. Figure 9(a) illustrates the static bed at an air velocity of 4 m/s, where air percolates through the bed without inducing particle movement. At air velocities of 8 and 12 m/s, the transitional stages between the static bed and spouting can be observed. Initially, a slight particle movement occurs at the base of the bed, as shown in Figure 9(b), where the air pushes the particles near the injection nozzle. This leads to the formation of a cavity in the particle bed, as illustrated in Figure 9(c). At velocities of 24 m/s, Figure 8 (d), and 30 m/s, Figure 9(e), the spout was fully developed. During this phase, the entire particle bed became fluidized, and a stable spout was formed, making it possible to clearly distinguish the characteristic regions of the spouted bed.

**Figure 9:**
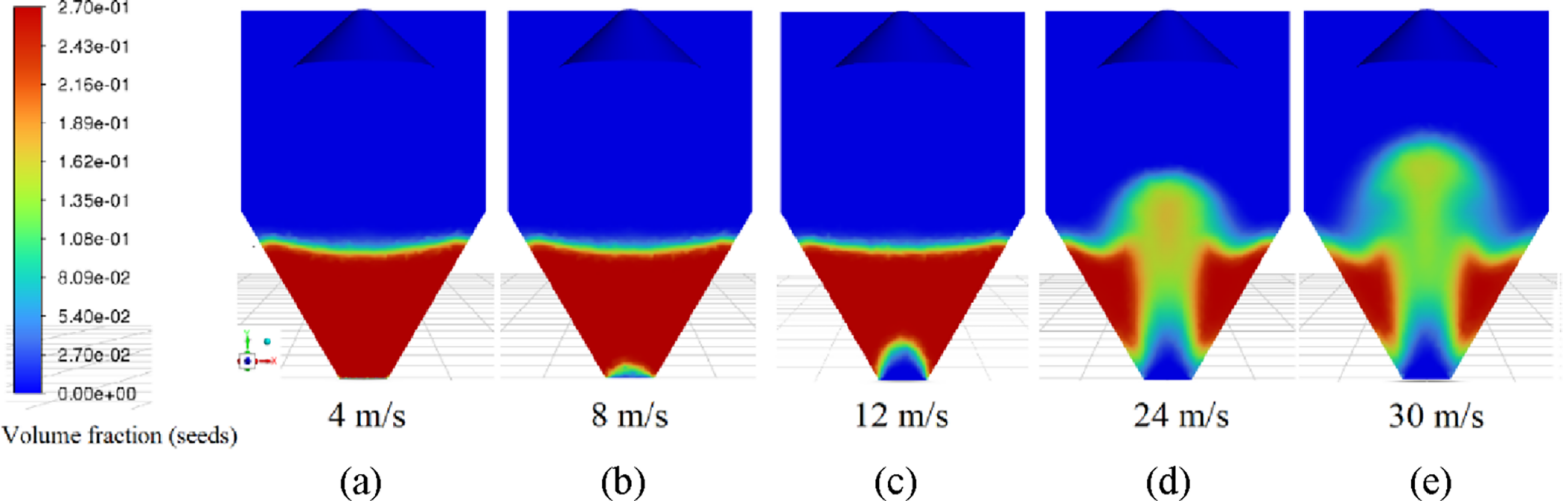
Volumetric fraction of solids for air inlet velocities of (a) 4, (b) 8, (c) 12, (d) 24, and (e) 30 m/s.

It is important to note that the simulation was unable to predict the typical behavior of the characteristic curve for the spouted bed, particularly regarding the pressure drop values as a function of air velocity. Although Figure 9 shows the transition of flow regimes leading to the establishment of a spout, this behavior does not correspond to the expected variation in pressure drop values presented in Figure 8. Figure 8 demonstrates a steady decrease in pressure drop values with increasing air velocity, making it impossible to identify the minimum spout velocity. A similar trend was observed in earlier spouted bed flow simulations using the TFM model, as reported by Batista et al.^34^

Batista et al.^35^ studied the fluid dynamics of conical spouted beds operating with sorghum grains both experimentally and through TFM (2D and 3D) and CFD-DEM simulations. The authors observed that CFD-DEM simulations were able to accurately predict the behavior of the spouted bed, allowing the detection of key stages of the process, such as bed transition and stable spout conditions. For the 2D and 3D TFM simulations, the fluid dynamic curves did not exhibit the typical expected behavior, making it impossible to identify the minimum spout velocity. To address this, the authors used solid volume fraction contour profiles.

##### Comparison of the Flow Regime with and without the Use of the Fountain Deflector

In order to analyze the impact of incorporating a fountain deflector on the fluid dynamics of the spouted bed, computational simulations were performed using bed geometries both with and without the deflector, at air velocities of 4, 15, 21, 30, 36, and 42 m s^–1^. The resulting solid volume fraction contours are presented in Figure 10.

**Figure 10:**
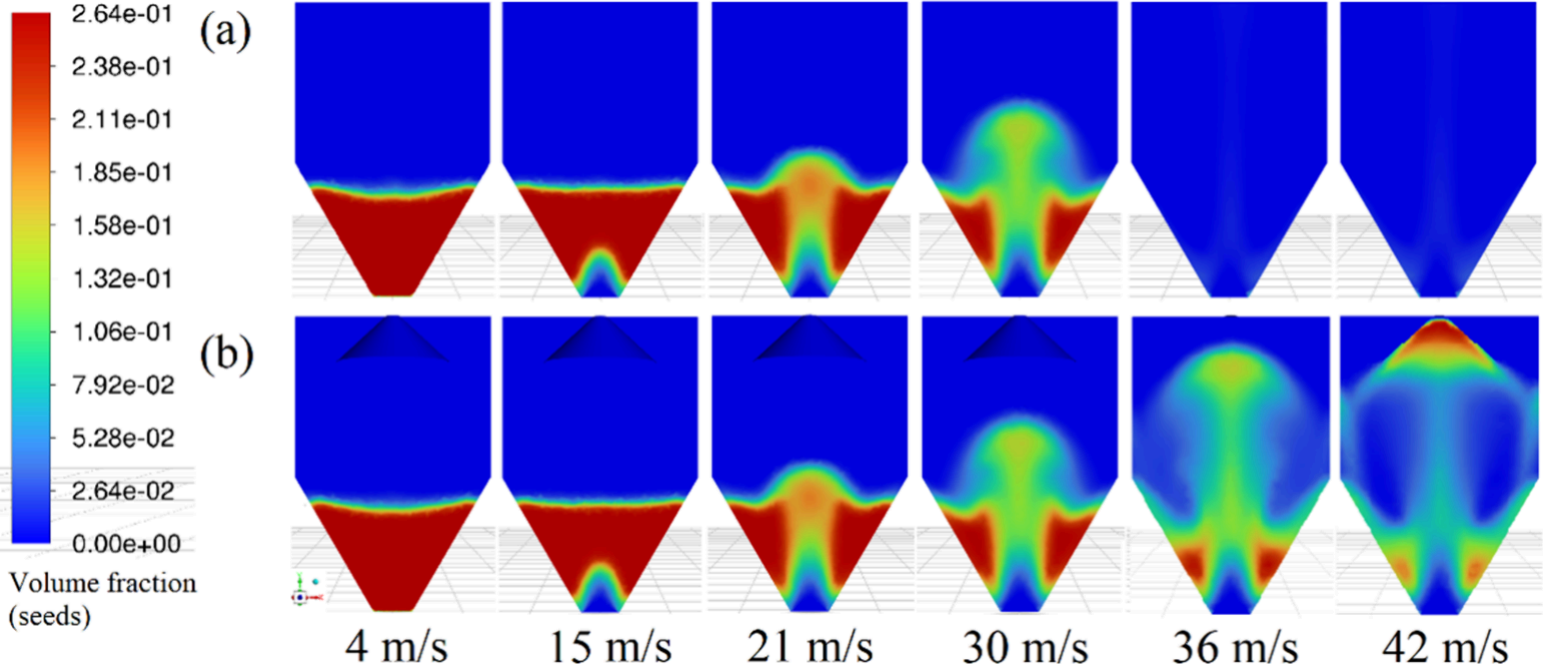
Solids volume fraction profiles for the flow in bed (a) without and (b) with fountain deflector.

The simulations for the geometry without the deflector showed the typical behavior expected from a spouted bed, with the spout forming at an air velocity of 21 m s^–1^, as shown in Figure 10(a). It is observed a static bed at an air velocity of 4 m s^–1^, where air percolates through the bed without inducing particle movement. At air velocity of 15 m s^–1^, a slight particle movement occurs at the base of the bed, where the air pushes the particles near the injection nozzle and leads to the formation of a cavity. At velocities of 21 m s^–1^ and 30 m s^–1^, the spout was fully developed. Comparable behavior was observed in the bed with fountain deflector, Figure 10(b), for air velocities up to 30 m s^–1^.

At higher air velocities, such as 36 m s^–1^, differences between the systems became evident. In the setup without fountain deflector, Figure 10(a), all seeds were carried out of the bed due to excessive entrainment. Meanwhile, the bed with deflector sustained a stable spout that reached its maximum height, restricted by the deflector, Figure 10(b). The fountain deflector clearly acts as a physical barrier—that is a wall that redirects particles back toward the lower region of the bed. It is worth noting that, while the source region is confined by the deflector, the annular and spouted regions are preserved. Higher increments in air velocity will result in particle accumulation regions near the sidewalls of the bed, as observed in the profile with the deflector at 42 m s^–1^.

It is important to state that although Figure 10 shows the transition of flow regimes leading to the establishment of a spout, the simulation was unable to properly predict the typical behavior regarding the pressure drop values as a function of air velocity. Data obtained showed a steady decrease in pressure drop values with increasing air velocity. Similar results were reported using TFM model simulations.^34,35^

## 3 Conclusions

This study investigated the influence of a fountain deflector, a device that has been rarely reported in the literature, on the fluid dynamics of a medium-scale spouted bed operating with dried papaya seeds.

The physical characterization of dried papaya seeds without the exotesta indicates that the particulate material has a high degree of sphericity, low apparent density, and a mean particle diameter of less than 5 mm. The seeds were classified within Group D of the Geldart diagram, which is associated with large and/or dense spoutable particles. The particle bed was found to exhibit low density and high porosity.

The fluid dynamic curves for the spouted bed, with and without the fountain deflector, were obtained. A comparison of these curves showed that the typical behavior expected for a spouted bed was preserved and the use of the deflector did not result in any significant changes in the fluid dynamic parameters

The fluid dynamic analysis conducted via CFD did not yield a curve representative of the expected spouted bed behavior. Nevertheless, the solid fraction contours revealed the characteristic stages of spout formation, as well as the particle retention effect induced by the fountain deflector. The deflector acts by limiting the height of the spout, redirecting particles back to the lower part of the bed and preventing them from being carried out of the equipment by the air.

Experiments conducted with the deflector positioned 0.06 m above the conical base and air velocities of 28 m s^–1^ or higher for 30 min resulted in a reduction in seed size confirming particle comminution at elevated air velocities.

Thus, the results indicate the potential of the fountain deflector to stabilize the regime in larger-scale beds, to reduce or eliminate particle elutriation, and to reduce particle size, enabling the expansion of spouted bed applications—even with materials that tend to be easily entrained or produce unstable flows and for systems where high gas flow rates are necessary.

## Symbols Used

*V*_*mj*_ [m s^–1^]: minimum spouting velocity

## Greek letters

Δ*P*_*max*_ [Pa]: maximum pressure drop
Δ*P* [Pa]: pressure drop
